# Effects of subject-specific professional knowledge and skills of physical education teachers on students’ learning progress

**DOI:** 10.1007/s12662-024-00992-0

**Published:** 2024-11-08

**Authors:** Matthias Wittwer, Roland Messmer, Jolanda Vogler

**Affiliations:** 1 Pädagogische Hochschule FHNW, Hofackerstrasse 30, 4132 Muttenz, Switzerland; 2 Institut für Bildungswissenschaften, Universität Basel, Hofackerstrasse 30, 4132 Muttenz, Switzerland

**Keywords:** EPiC-PE, Content Knowledge, Pedagogical Content Knowledge, Longitudinal study, Cascade model in physical education

## Abstract

Although the subject-specific professional knowledge of teachers is considered to be of central importance with regard to the learning performance of their students, there is only sparse empirical evidence on this issue. Moreover, current studies are primarily limited to the subject of mathematics. The aim of this article is to investigate the significance of subject-specific professional knowledge and skills of physical education (PE) teachers on the practical learning gains of their students based on a panel study. In order to measure both non-contextual knowledge and contextual skills on the teachers’ side, paper–pencil tests as well as video and text vignettes were used as test instruments. On the students’ side, practical sport skills were assessed in a pretest–posttest design to evaluate competence growth with regard to complex sports performance. An effect chain with mediation processes was postulated. Multilevel modeling analyzing 1669 students who were taught by 74 teachers could prove significant performance improvements for the students, but none of the four differentiated knowledge dimensions (content knowledge, non-contextual; pedagogical content knowledge, non-contextual; content knowledge, contextual; pedagogical content knowledge, contextual) of the teachers could explain these learning gains. Thus, the results are in line with various studies in mathematics and suggest that direct effects of teachers’ professional knowledge on students’ learning gains seem to be lost. The results therefore suggest that while basic and broad professional knowledge is required for instructional success, specific expertise in certain competence areas does not lead to additional learning success among students. Further research within the *EPiC-PE* study is needed to provide a more comprehensive picture of the relationships between professional knowledge and skills, classroom behavior, instructional processes, and learning outcomes.

**Supplementary Information:**

The online version of this article (10.1007/s12662-024-00992-0) contains supplementary material, which is available to authorized users.

## Introduction

It is widely accepted that teachers play a central role in providing good learning opportunities for students in order to promote their learning (e.g., Hattie, 2009). As a result, research efforts on the topic of teachers’ professional competence and, in line with the “knowledgeable teacher hypothesis” (Kunter et al., 2013, p. 806), on professional knowledge in particular have significantly increased in recent years (e.g., Krauss et al., 2017; Messmer et al., 2022). This focus has led to a significant improvement in the quality of such studies. Increasingly, they are not only based on self-assessments (subjective) or school grades (distal; e.g., Iserbyt, Ward, & Li, 2017) but more and more on standardized tests with the aim of capturing specific facets of competencies (objective and proximal; e.g., Wittwer et al. 2023).

On the students’ side, competence surveys are also becoming more reliable, moving beyond cross-sectional designs to increasingly address longitudinal research designs (e.g., Kunter et al., 2013). These developments in the quest to “trace the long route from teacher disposition to student learning” (Krauss et al., 2020, p. 312) have led to the possibility of increasingly detailed insights into the process of teaching subject-specific competencies.

Against this background, Krauss et al. (2020) conceived the so-called cascade model. This defines an integrative causal chain that integrates relevant teacher competence models – e.g., COACTIV (Baumert & Kunter, 2011) and competence as a continuum (Blömeke et al. 2015a) – typical causal models of teacher competence to teaching and learning goals (e.g., Helmke, 2015), models of teaching quality, and paradigms of teacher research. Taking increasingly more factors into account, first pathway models could be calculated, showing significant effects of teachers’ professional competencies on students’ learning outcomes (e.g., Blömeke, Jentsch, Ross, Kaiser, & König, 2022; Krauss et al., 2020; for an overview, see Yang & Kaiser, 2022).

While current research primarily addresses mathematics education, the state of research in other subjects is much less advanced. Thus, relevant studies for physical education (PE) are still pending. Accordingly, the adaptation of the cascade model to the subject of PE (Messmer et al., 2022) has not yet been empirically analyzed. One reason for this research gap can be seen in the fact that the development of suitable test instruments has only been advanced recently (e.g., Büchel et al., 2022; Heemsoth & Wibowo, 2020; Vogler, Messmer, & Allemann, 2017; Wittwer, 2021; Wittwer et al., 2023). This is despite the fact that the triad of professional knowledge defined by Shulman (1986, 1987)—*content knowledge* (CK), *pedagogical content knowledge* (PCK), *general pedagogical knowledge* (GPK)—is largely (i.e., with the exception of GPK) subject specific and, accordingly, must be examined from within the subject.

Against this background, the present paper aims to investigate the relationship between subject-specific professional knowledge and skills of PE teachers (CK and PCK) and the learning progress of their students. In line with current efforts in professional research (e.g., König, 2015), professional knowledge will be captured not only as dispositional (i.e., non-contextualized knowledge) but also contextualized as professional skills via hypothetical teaching scenarios.

## Theoretical framework

### Professional knowledge and skills

Following the *competence theory approach*, competence areas and knowledge dimensions are defined for the teaching profession which are necessary for mastering the tasks involved (Terhart, 2011). According to this approach, teachers are professional if they possess the most comprehensive competencies possible in the various areas of teaching requirements (diagnosing, assessing and advising, self-direction skills, etc.). The degree of professionalism is determined by the achievement of defined competence levels and by the effect of the teacher’s actions in the form of the greatest possible learning and experience gains for the students. The German-speaking discourse was particularly influenced by the competence model of the COACTIV study (Baumert & Kunter, 2011). This model specifies not only affective-motivational but also cognitive aspects that teachers must have in order to teach successfully. The focus of the present study is on this cognitive competence, but it does not exclude the fact that behind these cognitive competencies of teachers, especially in PE, there are also skills in sports practice.

Following the *cognitive perspective* on professional competence that has long been predominant in German-speaking professional discourse (e.g., Depaepe et al., 2013; Kaiser et al., 2017), central importance with regard to the acquisition of professional competence by their students is primarily attributed to *subject-specific professional knowledge* of teachers. It is thanks to Shulman (1986, 1987) in particular that professional research, long thought to be predominantly generic, is increasingly being examined in subject-specific terms. In addition to the *general pedagogical knowledge* (GPK) that every teacher should possess, regardless of the subject he or she teaches, good quality subject-specific teaching requires adequate *subject-specific professional knowledge* (CK and PCK). By CK, Shulman (1986) means adequate knowledge of the *facts* and *reasons* pertaining to the subject content. PCK comprises the ability to convey instructional content in a way that is appropriate to the addressee and knowledge about possible (mis)conceptions of students. While Shulman’s dimensioning in the field of CK has hardly been applied in theory and empiricism, studies in the field of PCK consistently build on this distinction (Depaepe et al., 2013). Subject-specific professional knowledge is understood as cognitive or dispositional knowledge. Primarily paper–pencil tests serve as psychometric test instruments for its assessment (Depaepe et al., 2013; Kaiser et al., 2017). Corresponding studies, especially in mathematics, could show that the areas CK and PCK, which are measured in this way, represent highly correlated but differentiable knowledge dimensions (e.g., Krauss et al., 2008).

However, the reduction of professional knowledge to purely dispositional, i.e., non-contextual aspects, which underlies the cognitive perspective, falls short. Professional knowledge (*knowing that*) cannot simply be transferred into practical skills (*knowing how*; Ryle, 2009). Teaching action is often characterized by intuitive and spontaneous decision-making without explicitly recalling knowledge (Bromme, 1992). Therefore, a *situated perspective* on teacher competence is receiving more and more attention. Following this approach, contextualized assessments of subject- and situation-specific professional knowledge and skills are increasingly sought (e.g., König, 2015). Thus, the shift in perspective that has long been called for in theoretical work, using predominantly video and text vignettes, is increasingly being implemented empirically (e.g., Blömeke et al., 2022; Kaiser et al., 2017; Vogler et al., 2017). A vital difference between the *knowledge* and *skills* of teachers is their proximity to observable behavior in the classroom. Whereas knowledge includes generalized cognitions that are not necessarily associated with a particular teaching situation, cognitive skills tend to be organized contextually (Putnam & Borko, 2000). Thereby, it is crucial to keep in mind that these skills have to be understood as cognitive abilities of teachers and should not be equated with observed behavior, i.e., performance (Neuweg, 2014). Teachers must cognitively perceive a specific instructional situation as relevant, interpret it accordingly, and finally decide how to act (PID model; Blömeke et al., 2015a). Although both the cognitive and the *situational perspective* share the underlying theoretical basis of expertise research from cognitive psychology, representatives of the situational perspective rarely refer explicitly to Shulman’s knowledge taxonomy. Rather, approaches such as the PID model just described serve as their theoretical foundation. These have been found to be interrelated due to their process-oriented nature, making them difficult to separate (Santagata & Yeh, 2016).

For PE, although sport didactics in the German-speaking discourse can look back on a long tradition of professional biographical (Miethling & Giess-Stüber, 2007) and casuistic (Lüsebrink et al., 2014) studies, research efforts to model professional knowledge of PE teachers have only recently been effectively advanced (e.g., Büchel et al., 2022; Heemsoth & Wibowo, 2020; Vogler et al., 2017). Corresponding studies can be located at different points on the competence continuum (Blömeke et al., 2015a; Vogler et al., 2018) and the majority point to the empirical separability of CK and PCK as well as the separability of individual dimensions within PCK (see Depaepe et al., 2013). The most recent study by Wittwer et al. (2023), which is based on the dataset of the present article, measures subject-specific professional knowledge (CK and PCK) as both non-contextualized knowledge and contextualized skills, in accordance with the cascade model in sports didactics (see Messmer et al., 2022). This showed that it is hardly possible to separate the individual dimensions due to strong correlations.

### Students’ learning progress

The assessment of students’ competencies has received considerable attention since the introduction of large-scale studies such as TIMSS or PISA at the turn of the millennium. However, these tests in students have so far primarily focused on the school’s core subjects (mathematics and language; Krauss et al., 2017). Although there are also initial test procedures for PE, in German-speaking countries these are mainly limited to motor and sports motor skills (e.g., Herrmann et al., 2019). This can be explained by the fact that especially for the so-called cgs sports (centimeters, grams, seconds) as well as for motor and technical contents, exact performance recordings are possible without great effort. However, the curriculum of the subject comprises much more than just these easily quantifiable content areas (e.g., Gogoll, 2020; Messmer, 2018) and, following the curricula in various countries (e.g., Lehrplan21 in German-speaking Switzerland), covers a wide range of competence areas (performing and dancing, playing, moving on equipment, etc.). In the English-speaking discourse, there are tests to assess tactical skills, particularly in the area of the *Teaching Games for Understanding* approach (e.g., Pill et al., 2023). However, with the *Game Performance Assessment Instrument* (Oslin et al., 1998), for example, such tests focus on holistic game observations and less on controllable and standardized exercises. Also, competencies in PE differ from competencies of the more cognitively accentuated subjects, as they are mainly expressed in body- and movement-related performances. Although cognitive competencies are also taught in PE, the focus is nevertheless on practical sports skills in the sense of tacit knowledge (see Polanyi, 1966).

### Relations between professional knowledge and learning progress

Following Helmke’s (2015) utilization-of-learning-opportunities model, students’ performance development is influenced by a wide range of factors. There are no isolated, simple, and stable dependence relationships between criteria of instructional success and characteristics of the teacher or the teaching. Teaching is to be understood as an offer for students, from which the intended effects do not necessarily result (constructivist learning approach). The scientific and didactical expertise of teachers can thus be identified as a key factor, which, however, has to be located within a complex structure of (potential) factors.

A pioneering role in the investigation of the implicit chain of effects or the cascade model (Krauss et al., 2020) again falls to the mathematical and scientific subjects. Thereby, it could be shown that the CK of teachers has no or only little predictive power for the learning performance of students (Baumert et al., 2010; Eisenberg, 1977)^1^. However, various studies (also for PE) show very strong relationships between CK and PCK (Büchel et al., 2022; Krauss et al., 2017; Wittwer et al., 2023). “Without a deeper understanding of content, teachers will be unable to teach meaningful outcomes in physical education” (Ward, 2013, p. 438). PCK, on the other hand, appears to be beneficial for cognitively challenging teaching and, more importantly, to positively influence student achievement progress^2^. The impact of this difference in explanatory power between the CK and PCK of teachers on student achievement may be explained by the fact that PCK, in contrast to CK, has so far mostly been assessed by tests that are more strongly based on problem-based instructional scenarios (Baumgartner, 2018). For example, Shulman (1986, 1987) already described PCK in very behavioral terms. Corresponding studies are accordingly increasingly based on situated tests. Instead of pedagogical content knowledge, aspects of skills and, thus, among other things, *implicit* knowledge, are often already measured, which seems to lead to a higher predictive validity than in the case of CK. Thus, according to Bromme (2008), correlations between CK and teaching success particularly are not evident when CK is captured only by characteristics of university education and away from context. This assumption can also be supported by the fact that PCK seems to be more strongly related to situation-specific skills of teachers (PID) than CK in several studies (e.g., Blömeke et al., 2022).

Different theories also exist on the question of whether non-contextualized professional knowledge or contextualized ability is more important for students’ learning effects. On the one hand, theoretical knowledge can be seen as more *distal* and situational skills as more *proximal* in terms of proximity to student learning gains. Therefore, it can be hypothesized that contextualized skills have a closer connection to effects on the students’ side than non-contextualized knowledge (e.g., Neuweg, 2014). On the other hand, it can also be argued that situationalized testing, while more useful for understanding what teachers are thinking in the classroom, is less useful for predicting student performance. This would imply that contextualized skill tests are less effective at predicting student learning growth (see Krauss et al., 2020). Recently, Blömeke et al. (2022) and Krauss et al. (2020) attempted to clarify this ambiguity through empirical studies and reported contrary findings. While Krauss et al. (2020) identified non-contextualized PCK as the only construct with (small) positive effects on aspects such as cognitive activation (0.22*^99^) and learning support (0.21*), Blömeke et al. (2022) proved small positive effects on students’ learning performance only for contextualized skills (0.12**).

To date, such correlations have only been demonstrated in the field of mathematics (Yang & Kaiser, 2022). Nevertheless, in other subjects (including PE), positive correlations are often (implicitly) assumed between subject-specific professional knowledge or skills of teachers and the performance of their students. Empirical evidence for this, however, remains lacking.

## Research questions and hypotheses

We empirically specify a path model based on the EPiC-PE data that takes into account three of the five pillars of the cascade model of sport didactics (see Messmer et al., 2022; namely 1, 3, and 5). Accordingly, following the competence continuum approach of Blömeke et al. (2015a), we analyze the chain of effects from (1) dispositional subject-specific professional knowledge to (3) situation-specific knowledge and skills to (5) students’ learning progress in PE. In doing so, the superordinate research question to be answered is to what extent the subject-specific professional knowledge and skills of PE teachers predict students’ learning progress.

To answer this question and in the course of specifying the cascade model, the interrelationships between subject-specific professional knowledge and skills will also be clarified. Following the findings of Wittwer et al. (2023), significant correlations between the two dimensions of non-contextual professional knowledge (CK non-contextual [CK-N] and PCK non-contextual [PCK-N]) as well as between the dimensions of contextual professional skills (CK contextual [CK-C] and PCK contextual [PCK-C]) and medium to large effects of the former on the latter can be assumed. According to Wittwer et al. (2023), smaller correlations can generally be expected with contextualized PCK (PCK-C).

On this basis, we will then address the question of what knowledge or skills PE teachers need to have in order to foster student learning gains. Following Krauss et al. (2020) and considering the partly contrary results of Krauss et al. (2020) and Blömeke et al. (2022), it is difficult to predict the results of this study. However, an obvious hypothesis might be that cognitive ability as a proximal predictor may forecast student achievement gains with a greater effect size than the distal predictor of teachers’ dispositional knowledge (see Fishbein et al., 2001). Nevertheless, it can be assumed that dispositional knowledge is a necessary requirement for developing contextualized skills. Accordingly, it can be assumed that teachers’ skills are an “essential mediator” between teachers’ knowledge and their teaching (Meschede et al., 2017, p. 159).

## Methodology

### Context of the present study: *EPiC-PE*

This article is part of the EPiC-PE study funded by the Swiss National Science Foundation (see Messmer et al., 2022). The study aims to investigate interdependencies and effects of professional competencies of PE teachers on teaching and students. Thus, the research questions presented in this study single out one aspect of the overall study for closer examination.

EPiC-PE is a panel study with two measurement timepoints and an intervening teaching unit. In addition to the assessment of professional competencies of PE teachers, the development of student performance was recorded in a pretest–posttest design. For the teaching units, the learning objectives based on Lehrplan21 and the available teaching time of 12 lessons of 45 min each (around 6 weeks) were specified. Standardization with respect to a technical and tactical (TT) and a technical and aesthetical competence area (TA) based on Messmer’s (2018) subject model allows comparisons between teachers and between classes, respectively, as well as a content-based link between teachers’ competencies, teaching, and student performance. The two areas of competence, TT and TA, which are far apart in Messmer’s subject model, were chosen intentionally in order to cover PE as broadly as possible despite a narrow subject conception.

### Constructs and instruments

The study was preceded by a test development and validation phase as well as by a pilot test. More detailed explanations of this process and of the individual survey instruments can be found in Wittwer (2021), Wittwer et al. (2023), and Messmer et al. (2022). In the following, the focus is primarily on the reporting of test quality criteria. A more detailed derivation will be described for the testing of students’ practical sport competencies, as these have not been published before. The test quality criteria of the students’ tests can be found in the Results section.

#### Paper–pencil tests (CK non-contextual and PCK non-contextual)

For the assessment of non-contextual CK, 21 MC items in the area TT and 28 MC items in the area TA were used. The items aimed for a deeper understanding of the content of the secondary school curriculum (see Wittwer, 2021). Reliabilities were 0.78 for TT and 0.83 for TA with respect to Cronbach’s α. For the non-contextual PCK (i.e., knowledge), the reliabilities were 0.71 for TT with 19 items used and 0.89 for TA with 29 items. To account for the complexity of the items, partial credit models (Masters, 1982) were specified in the data analysis, which also allowed for partially correct solutions (see Wittwer et al., 2023).

#### Video vignettes (CK contextual)

To assess contextual CK (i.e., skills), a test instrument developed by Wittwer (2021) was used with six video vignettes showing lower secondary students performing movement tasks within the two content areas (three on TT, three on TA). The movement executions showed various deficiencies, which were to be (1) recognized and (2) justified (MC format). In addition, rating tasks were used (3) to assess the quality of possible movement corrections. Quasi-pair comparisons with expert assessments allowed judgments to be made about the quality of the participants’ answers (see Tepner & Dollny, 2014). Partially correct answers were possible for all items (partial credit). Cronbach’s α was 0.86 for the TT part for 23 items and 0.66 for the TA part for 9 items (Wittwer et al., 2023).

#### Text vignettes (PCK contextual)

For the contextual PCK (i.e., skills), 11 (5 TT and 6 TA) text vignettes describing situations in PE with critical incidents were used. The vignettes could be adopted from Vogler et al. (2017). As an innovation, a rating format was used here rather than an open response format for reasons of research economy. Thus, for each vignette, four to six given alternative actions (items) are to be evaluated with regard to their quality. Quasi-pair comparisons with expert norms in a partial credit procedure were also used for evaluation. Cronbach’s α was 0.51 for the TT part with 5 (very complex) items and 0.68 for the TA part with 7 items (see Wittwer et al., 2023). This selected test procedure based on vignettes and rating scale tasks enables objective and efficient evaluation of a didactic large-scale test (see Tepner & Dollny, 2014).

#### Student achievement in PE

New instruments had to be developed in order to meet the requirement of recording not only sports motor skills but also complex sporting actions. In the construction of the test items, the EPiC-PE project was guided by the two selected competence dimensions and the learning objectives of the Lehrplan21 valid in German-speaking Switzerland, which were given to the teachers as a basis for planning. For the area TT, three test items (technique course basketball, pick’n’roll basketball, pick’n’roll handball) and for the area TA, four test items (horizontal bar, floor routine, dance reproduction, dance variation) were developed. The tasks were described in a detailed test administrator manual and were used by trained test administrators as a basis for the tests. The students’ performances were videotaped and rated by raters based on rating manuals (5–8 raters per test area). The tests were composed of 3–8 items per test domain in partial-credit or dichotomous form. The test items and rating manuals were reviewed during test development by means of a multi-stage expert survey with experts in the respective disciplines (*N* = 10).

#### Control variables

In order to adequately identify biases in the assessment of the importance of professional knowledge on students’ learning gains, possible confounding variables should be controlled. Various studies in the field of educational research indicate that especially prior achievement, student background, school context, and school type were significantly related to outcomes (e.g., Baumert et al., 2010). For PE as a less cognitively accentuated school subject, there are hardly any studies so far. However, it can be assumed that practical sport performance is less related to cognitive criteria than to extracurricular physical activities and previous sport motor experiences. Thus, PE is characterized in particular by the fact that contents of the lessons are also addressed outside of school in leisure time and in sports clubs. As a consequence, prior experiences in sport motor skills were taken into account as control variables in the form of subjective statements by the students on their involvement in the respective sports in their free time.

### Pilot study

The practical feasibility of the tests of the students’ practical sports skills was tested in a pilot phase with 11 school classes and 173 students and optimized on this basis. The rating manuals were checked for applicability with the support of 18 raters and revised if necessary.

The pilot study to test the professional knowledge and skills of the teachers included a sample of 201 (143 on TT and 58 on TA) prospective teachers of PE at the secondary level, who are completing their education at Swiss and German universities. The pilot phase confirmed mostly satisfactory parameters regarding reliability but also offered a basis for further optimization, especially regarding construct validity. Based on this, various items were adapted or, if necessary, excluded from the test. No fixed limits were set a priori. Rather, the data provided an opportunity for critical examination of the items and their possible modification (Wittwer et al., 2023).

### Sample of the main study

Seventy-six lower secondary school PE teachers who teach at schools in the German-speaking part of Switzerland participated in the main study. The female (39.5%) and male (60.5%) teachers had a mean age of 40.5 years (standard deviation [SD] = 10.2) and a mean of 13.8 years of professional experience (SD = 9.8). To examine the construct validity of the test instruments used to assess subject-specific knowledge and skills with respect to the underlying model, 105 prospective PE teachers were added to the sample (of which 44.8% were female students and 55.2% were male students; mean age: 26.5 years; Wittwer et al., 2023). The 76 participating PE teachers taught a total of 1669 students (48.8% female) in 90 classes. Teachers were randomly divided between the two skill areas of TT and TA, with a focus on TT. Participation in the study was voluntary for the teachers. Therefore, possible bias due to self-selection must be assumed.

### Data collection, processing, and analysis

A few weeks before the start of the study, the teachers received the learning objectives that they had to work on with their class in a time window of 12 lessons (approximately 6 weeks). The knowledge tests of the teachers were conducted before the beginning of the lesson using the online survey software Unipark (Tivian XI GmbH, Hürth, Germany). For this purpose, they were given a time window of 90 min during which the pretest was conducted with their class. As a result, the teachers were not able to see this test being performed. Approximately 6 weeks later the posttest was carried out.

The students’ practice tests were videotaped and assessed by trained raters. To ensure interrater reliability, some classes were initially assessed in parallel during multiple runs by five to eight raters until there was sufficiently high agreement (Krippendorff’s ⍺ ≥ 0.8; Hayes & Krippendorff, 2007). Subsequently, the classes were divided among the individual raters.

**Fig. 1:**
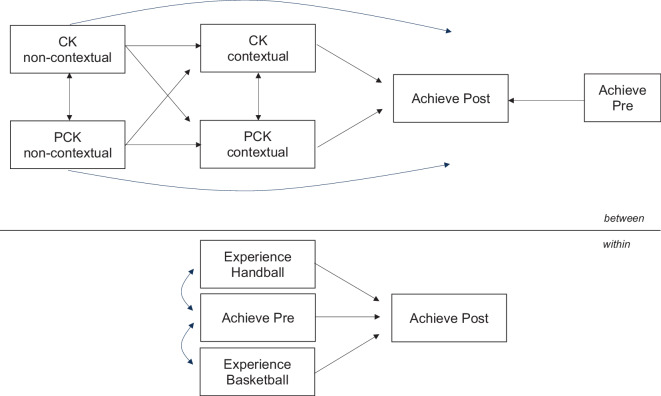
Mediation model for the effect of subject-specific professional knowledge on contextualized subject-specific skills and the learning progress of students in the subject of PE under the control of sport-specific previous experiences (*CK* content knowledge, *PCK* pedagogical content knowledge)

To test our hypotheses about the chain of effects, a series of two-level random-intercept mediation models were computed with students on the within and with teachers on the between level (see Fig. 1). After checking the reliability of the individual dimensions, the performance of the students and the individual dimensions of professional knowledge and skills of the teachers were considered as manifest variables due to the reduced sample size (in contrast to the previous studies, the data of the trainee PE teachers could not be included in the present study). Missing data were treated with the maximum likelihood procedure with complete information. To examine the robustness of our model, we applied several alternative approaches. The differences were negligible. All analyses were performed using the statistical software package Mplus version 8 (Muthén & Muthén, 2017). Direct and indirect effects were estimated. Following Cohen (1992), coefficients around 0.10 were interpreted as weak, around 0.30 as moderate, and around 0.50 or higher as strong direct effects.

## Results

### Descriptives results and preliminary examinations

The assumed dimensionality of facets of professional knowledge and skills was tested and reported in Wittwer et al. (2023). The adopted structure (CK non-contextual, PCK non-contextual, CK contextual, PCK contextual) showed good local and global fit (TT: χ^2^ (14) = 19.64, *p* = 0.14, CFI = 0.99, RMSEA = 0.05, SRMR = 0.03; TA: χ^2^ (14) = 24.68, *p* = 0.04, CFI = 0.97, RMSEA = 0.1, SRMR = 0.04). However, a clear differentiation of the four assumed dimensions was limited due to the high correlations. The investigations showed, however, that differences between the dimensions do exist. In addition, as already described, a partial cohort had to be excluded for the present evaluations in comparison to the evaluations of Wittwer et al. (2023), since only teaching staff who participated in the study together with their classes can be taken into account. Comparing the performance of these cohorts reveals large differences in the subject-specific professional knowledge and skills of teaching and pre-service PE teachers. Teachers significantly outperformed prospective teachers in all dimensions surveyed (except: TA CK contextual) and with moderate to strong effects (Cohen’s d: 0.48–0.98).

While descriptive statistics on teachers’ subject-specific professional knowledge and skills have already been reported above—and in more detailed form in Wittwer et al. (2023)—corresponding results on testing students’ sports practical skills can be found in Table 1. In spite of the ambitious attempt to record complex sporting activities and the broad coverage of the competence areas recorded, the tests carried out resulted in acceptable to good reliabilities. When comparing the two test times (t1–t2), significant improvements in performance can be seen for both competence areas (Table 2). This shows that the PE classes conducted by the PE teachers had a positive influence on all partial performances of the students. The effect sizes according to Cohen (1992) are in a weak (0.16) to strong range (0.78).

**Table 1:** Descriptive statistics and internal consistencies (Cronbach’s alpha) of students’ recorded sports practical competencies

Construct (no. items)	*N*	M	SD	Min	Max	Cronbach’s α
TT–t1 (19)	598	17.48	4.18	6	31	0.60
TT–t2 (19)	448	20.44	4.13	7	33	0.58
TA–t1 (9)	358	20.99	10.51	1	53	0.84
TA–t2 (9)	286	27.87	10.89	1	53	0.86

*N* sample size, *M* mean, *SD* standard deviation, *t1/t2* measurement timepoints, *TT* technical and tactical competence area, *TA* technical and aesthetical competence area

**Table 2:** Paired-sample *t*-test comparing the performance of the students at t1 and t2

Dimension	Test	*N*	M (SD)	*t*	*d*
TT	Technique course	493	−0.60 (2.18)	−6.08***	0.27
	Pick’n’roll basketball	463	−1.00 (3.18)	−6.67***	0.31
	Pick’n’roll handball	458	−0.58 (3.72)	−3.34***	0.16
TA	Horizontal bar	288	−1.33 (2.94)	−7.67***	0.45
	Floor routine	288	−0.52 (1.83)	−4.88***	0.29
	Dance	299	−4.72 (6.03)	−13.54***	0.78

*TT* technical and tactical competence area, *TA* technical and aesthetical competence area, *N* sample size, *M* mean, *SD* standard deviation, *t* t-value, *d* Cohenʼs d effect sizes

*p ≤ 0.05; **p ≤ 0.01; ***p ≤ 0.001

### Bivariate correlations of professional knowledge dimensions with other factors

Table 3 shows the correlations between the recorded knowledge dimensions and other biographical factors. It shows, among other things, that the workload of the teachers in the field of sport has a positive correlation with several dimensions. In addition, the training of the teachers also seems to be important in this respect. Thus, teachers who have a teaching diploma for upper secondary level have a greater knowledge than their professional colleagues with a degree for lower secondary level. Significant positive correlations were also found between several dimensions of professional knowledge and membership and coaching in sports clubs in the corresponding skill areas. In line with gender stereotypes, male PE teachers also perform better in sports games (TT) and female PE teachers in aesthetic sports (TA). Overall, no clear tendency can be identified as to whether CK or PCK or contextual or non-contextual knowledge areas show clearer correlations with the biographical factors. In need of explanation is the relatively clear negative correlation between professional knowledge/skills and the final grade in the secondary school diploma in the field of TT.

**Table 3:** Bivariate correlations of the four dimensions of professional knowledge and skills with biographical factors (Pearson product-moment correlations)

		CK‑N	PCK‑N	CK‑C	PCK‑C
TT	Sex (1: female; 2: male)	0.29*	0.20	0.36*	0.08
	Workload (PE)	0.35*	0.15	0.17	0.18
	Final grade Matur (Swiss Abitur)	−0.36*	−0.38**	−0.39**	0.00
	Membership in a sports club of the invasion games	0.29*	0.21	0.27*	0.13
	Coaching in a sports club of invasion games	0.42**	0.17	0.40**	0.15
TA	Sex (1: female; 2: male)	−0.18	0.08	−0.21	−0.30
	Workload (PE)	0.40*	0.42*	0.20	0.15
	Teaching diploma for higher secondary level	0.49*	0.39	0.13	0.26
	Final grade Matura (Swiss Abitur)	0.06	0.02	0.04	0.10
	Membership in a gymnastics club	0.07	−0.11	0.12	0.10
	Membership dance studio/club	0.02	0.44*	0.58**	−0.09
	Coaching license gymnastics	0.49*	0.36	0.33	0.40*
	Coaching license dance	0.40*	0.26	0.22	0.21

Technical and tactical competence area (TT): *N* = 47–49

Technical and aesthetical competence area (TA): *N* = 26

*CK‑N* content knowledge non-contextual, *PCK‑N* pedagogical content knowledge non-contextual, *CK‑C* content knowledge contextual, *PCK‑C* pedagogical content knowledge contextual, *PE* physical education

*p ≤ 0.05; **p ≤ 0.01; ***p ≤ 0.001

### Effects of subject-specific professional knowledge and skills of PE teachers on students’ learning progress

The intraclass correlation of the students’ PE performance was 0.42 for TT and 0.18 for TA, indicating significant differences in performance between classes, especially for TT. For both TT and TA, the implemented instructional unit caused a significant increase in performance. The predictive effect of student performance at the first measurement timepoint with respect to the measurement timepoint after the instructional unit was moderate to high at the between level (TT: β = 0.37; TA: β = 0.78; see M1 in Tables 4 and 5). However, only in TA could a significant variance of the posttest be explained by results of the pretest (*R*^*2*^ = 0.61). The students’ prior experience in the respective sports showed a significant positive correlation with the performance at the first timepoint (TT: 0.13**; TA: 0.20*** and 0.21***; see M2 in Tables 4 and 5) but no effects on the posttest results. Contrary to our hypothesis, no significant effects of professional knowledge and skills on students learning progress were observed—neither for TT (CK-N: β = −0.12; PCK-N: β = 0.07; CK-C: β = 0.11; PCK-C: β = 10) nor for TA (CK-N: β = 0.11; PCK-N: β = −0.32; CK-C: β = −0.22; PCK-C: β = 0.16; see M3).

**Table 4:** Relationship between teachers’ professional knowledge and students’ learning progress in TT (two-level random intercept model; standardized coefficients, standard errors)

	M1			M2			M3			M4		
Fixed effects	Est	SE	*p*-value	Est	SE	*p*-value	Est	SE	*p-*value	Est	SE	*p-*value
**Within level (*N*)**	730	–	–	1020	–	–	896	–	–	1087	–	–
*Student achievement t2 on*												
Student achievement t1	0.30	0.05	< 0.001	0.28	0.05	< 0.001	0.29	0.05	< 0.001	0.28	0.05	< 0.001
Experience basketball	–	–	–	0.03	0.05	0.56	0.02	0.05	0.73	0.03	0.05	0.56
Experience handball	–	–	–	0.10	0.05	0.04	0.09	0.05	0.06	0.10	0.05	0.04
*Student achievement t1 with*												
Experience basketball	–	–	–	0.13	0.04	0.002	0.14	0.05	0.002	0.13	0.04	0.002
Experience handball	–	–	–	0.13	0.04	0.001	0.14	0.04	0.001	0.14	0.04	0.001
*R*^2^	0.09	0.03	0.001	0.10	0.03	0.001	0.10	0.03	0.001	0.10	0.03	0.001
**Between level (k)**	37	–	–	55	–	–	48	–	–	55	–	–
*Student achievement t2 on*												
Student achievement t1	0.39	0.15	0.011	0.41	0.15	0.007	0.46	0.15	0.002	0.43	0.15	0.005
CK‑N	–	–	–	–	–	–	−0.12	0.24	0.60	−0.12	0.24	0.60
PCK‑N	–	–	–	–	–	–	0.07	0.22	0.74	0.08	0.22	0.72
CK‑C	–	–	–	–	–	–	0.11	0.25	0.67	0.11	0.26	0.68
PCK‑C	–	–	–	–	–	–	0.10	0.16	0.52	0.10	0.16	0.55
*Teachers CK‑C on*												
CK‑N	–	–	–	–	–	–	–	–	–	0.43	0.11	< 0.001
PCK‑N	–	–	–	–	–	–	–	–	–	0.43	0.11	< 0.001
*Teachers PCK‑C on*												
CK‑N	–	–	–	–	–	–	–	–	–	0.22	0.17	0.19
PCK‑N	–	–	–	–	–	–	–	–	–	−0.04	0.17	0.84
CK‑N with PCK‑N	–	–	–	–	–	–	–	–	–	0.58	0.10	< 0.001
CK‑S with PCK‑C	–	–	–	–	–	–	–	–	–	0.10	0.58	0.48
CK‑N_ind_ via CK‑C	–	–	–	–	–	–	–	–	–	0.05	0.11	0.68
CK‑N_ind_ via PCK‑C	–	–	–	–	–	–	–	–	–	0.02	0.04	0.56
CK‑N_tot_	–	–	–	–	–	–	–	–	–	0.07	0.11	0.56
PCK‑N_ind_ via CK‑C	–	–	–	–	–	–	–	–	–	0.05	0.11	0.68
PCK‑N_ind_ via PCK‑C	–	–	–	–	–	–	–	–	–	−0.03	0.02	0.85
PCK‑N_tot_	–	–	–	–	–	–	–	–	–	0.04	0.11	0.71
*R*^*2*^* CK‑C*	–	–	–	–	–	–	–	–	–	0.57	0.10	< 0.001
*R*^*2*^* PCK‑C*	–	–	–	–	–	–	–	–	–	0.04	0.06	0.46
*R*^*2*^* student achievement t2*	0.15	0.14	0.20	0.17	0.12	0.18	0.23	0.15	0.11	0.23	0.14	0.10

*CK‑N* content knowledge non-contextual, *PCK‑N* pedagogical content knowledge non-contextual, *CK‑C* content knowledge contextual, *PCK‑C* pedagogical content knowledge contextual

**Table 5:** Student learning gains and relationship between teacher professional knowledge and student learning gains in TA (two-level random intercept model; standardized coefficients, standard errors)

	M1			M2			M3			M4		
Fixed effects	Est	SE	*p-*value	Est	SE	*p-*value	Est	SE	*p-*value	Est	SE	*p-*value
**Within level (*N*)**	375	–	–	469	–	–	442	–	–	535	–	–
*Student achievement t2 on*												
Student achievement t1	0.76	0.03	< 0.001	0.74	0.03	< 0.001	0.74	0.03	< 0.001	0.74	0.03	< 0.001
Experience gymnastics	–	–	–	0.01	0.04	0.86	0.01	0.04	0.85	0.00	0.04	0.96
Experience dance	–	–	–	0.08	0.04	0.04	0.09	0.04	0.03	0.08	0.04	0.04
*Student achievement t1 with*												
Experience gymnastics	–	–	–	0.20	0.05	< 0.001	0.20	0.05	< 0.001	0.20	0.05	< 0.001
Experience dance	–	–	–	0.21	0.05	< 0.001	0.21	0.05	< 0.001	0.21	0.05	< 0.001
*R*^2^	0.58	0.04	< 0.001	0.58	0.04	< 0.001	0.58	0.04	< 0.001	0.58	0.04	< 0.001
**Between level (k)**	24	–	–	25	–	–	23	–	–	26	–	–
*Student achievement t2 on*												
Student achievement t1	0.78	0.11	< 0.001	0.74	0.14	< 0.001	0.74	0.14	< 0.001	0.78	0.12	< 0.001
CK‑N	–	–	–	–	–	–	0.11	0.25	0.67	0.10	0.23	0.66
PCK‑N	–	–	–	–	–	–	−0.32	0.25	0.21	−0.30	0.23	0.20
CK‑C	–	–	–	–	–	–	−0.22	0.18	0.22	−0.20	0.16	0.22
PCK‑C	–	–	–	–	–	–	0.16	0.21	45	0.16	0.19	0.42
*Teachers CK‑C on*												
CK‑N	–	–	–	–	–	–	–	–	–	0.11	0.24	0.64
PCK‑N	–	–	–	–	–	–	–	–	–	0.36	0.23	0.12
*Teachers PCK‑C on*												
CK‑N	–	–	–	–	–	–	–	–	–	0.35	0.21	0.10
PCK‑N	–	–	–	–	–	–	–	–	–	0.31	0.21	0.15
CK‑N with PCK‑N	–	–	–	–	–	–	–	–	–	0.66	0.11	< 0.001
CK‑N with PCK‑C	–	–	–	–	–	–	–	–	–	−0.07	0.20	0.74
CK‑N_ind_ via CK‑C	–	–	–	–	–	–	–	–	–	−0.02	0.05	0.66
CK‑N_ind_ via PCK‑C	–	–	–	–	–	–	–	–	–	0.06	0.08	0.47
CK‑N_tot_	–	–	–	–	–	–	–	–	–	0.14	0.22	0.54
PCK‑N_ind_ via CK‑C	–	–	–	–	–	–	–	–	–	−0.07	0.08	0.34
PCK‑N_ind_ via PCK‑C	–	–	–	–	–	–	–	–	–	0.05	0.07	0.48
PCK‑N_tot_	–	–	–	–	–	–	–	–	–	−0.02	0.11	0.83
*R*^2^ CK‑C	–	–	–	–	–	–	–	–	–	0.19	0.14	0.17
*R*^2^ PCK‑C	–	–	–	–	–	–	–	–	–	0.35	0.15	0.02
*R*^2^ student achievement t2	0.61	0.18	< 0.001	0.54	0.20	0.008	0.68	0.19	< 0.001	0.72	0.17	< 0.001

*CK‑N* content knowledge non-contextual, *PCK‑N* pedagogical content knowledge non-contextual, *CK‑C* content knowledge contextual, *PCK‑C* pedagogical content knowledge contextual

Next, the effects of professional knowledge and skills were examined based on the cascade model in sports didactics (see Fig. 1 and M4 in Tables 4 and 5). Thereby, positive correlations and effects between the assumed areas of professional knowledge and skills are (almost) consistently found for both TT and TA. Highly significant correlations can be found especially between the two dimensions of non-contextual knowledge (TT: *r* = 0.58; TA: *r* = 0.66). Both non-contextual dimensions also show weak to moderate positive effects on contextualized CK—in the TT domain these effects are highly significant (0.11 to 0.43***). Contextualized PCK, on the other hand, can only be explained to a limited extent by the other dimensions of professional knowledge and skills (β between −0.07 and 0.35). The indirect effects presented in model 4 also confirm the previously noted finding that no dimensions of professional knowledge and skills appear to have significant effects on students’ learning progress.

## Discussion

The aim of the present study was to model the effect of subject-specific professional knowledge and skills of PE teachers on the learning progress of their students with regard to practical sport competencies. For this purpose, paper–pencil tests as well as video and text vignettes were used as test instruments on the side of the teachers in order to capture contextual skills in addition to non-contextual knowledge. In contrast to previous studies, the dimensions CK and PCK were analyzed separately in the contextual assessment (Wittwer et al., 2023). On the side of the students, practical sport tests were carried out at two measurement timepoints—with a teaching unit in between—to record the increase in competence with regard to complex sportive tasks. Significant improvements in performance (M1) and correlations with previous sport practice experience (M2) were found in both content areas (TT and TA). In a further empirical step, the influence of subject-specific professional knowledge and skills (CK‑N, PCK‑N, CK‑C, PCK-C) on students’ learning gains was investigated using path models. The four-predictor models showed no significant effects (M3). Also, based on the path model shown in Fig. 1, no significant indirect effects of non-contextual knowledge via contextual skills on student achievement could be demonstrated (M4). Thus, the intraclass correlations found cannot be explained by effects of PE teachers’ professional knowledge and skills. Therefore, the present study can neither confirm the “knowledgeable teacher hypothesis” (Kunter et al., 2013, p. 806) nor the assumption that video and text vignettes capture the performance of teachers’ knowledge better than paper–pencil tests (as emphasized especially by proponents of the situational perspective, e.g., Kaiser et al., 2015). However, the results are largely in line with previous empirical studies in mathematics, which could not prove any or only weak systematic relationships between teachers’ professional knowledge and students’ learning progress (Baumert et al., 2010; Blömeke et al., 2022; Krauss et al., 2020; for an overview, see Yang & Kaiser, 2022), indicating that corresponding effects seem to get lost in the assemblage of supply and benefit (see Helmke, 2015) on “the long route from teacher disposition to student learning” (Krauss et al., 2020, p. 312). Moreover, inconsistent results in the aforementioned studies point to complex relationships that do not seem to be universally valid.

The assumption of positive correlations between the professional knowledge and skill dimensions as well as positive effects of explicit knowledge on situation-specific skills could be largely confirmed with the results in model 4. However, the correlations are in significantly lower ranges than expected from the structural analyses of Wittwer et al. (2023). This change is primarily due to the fact that the cohort of university students could not be taken into account in the current analyses, and thus deeper correlations seem to be present among teachers. As a consequence, the very good fit between item difficulty and person ability reported by Wittwer et al. (2023 also seems to have shifted slightly, as the tested teachers have significantly more knowledge and skills than the students examined in the EPiC-PE study (see “Descriptives results and preliminary examinations”). This also reflects corresponding comparisons in PE in Vogler et al. (2017). The resulting argument that professional experience positively influences teachers’ professional knowledge and skills is also underlined by the finding that teachers with a larger workload have better professional knowledge (Table 3). However, this conclusion is somewhat relativized by possible biases in the data due to self-selection of teachers (participation for teachers was voluntary). Either way, in view of the very weak effects of professional knowledge and skills on students’ learning performance, the hypothesis can be formulated that teachers must have a basic and broadly defined professional knowledge, but that above a certain level, deeper expertise in individual areas of competence no longer seems to lead to greater learning success on the part of the students. Thus, the teachers we tested may have known and been able to do too much to confirm our hypotheses. In this sense, Begle (1972) and Eisenberg (1977) already assumed that above a certain threshold, no correlations between teacher knowledge and student performance can be detected.

For the education of future PE teachers, one could conclude from this that a transfer of in-depth knowledge and a separation of subject science and subject didactics in the education of PE teachers is therefore not necessary. However, this return to a related model of teacher education as a master apprenticeship seems premature. As knowledge utilization research shows, the epistemological process of knowledge acquisition must be separated from the application of this knowledge (Bonß, 2001). This is especially important to keep in mind when it comes to professional knowledge, because there is a blending of slow and fast thinking (Kahneman, 2011).

The results also show that the cascade model formulated by Krauss et al. (2020) for PE needs to be questioned to some extent. For example, the model of a linear effect of teacher professional knowledge and skills on student learning outcomes for PE could not be confirmed. The amalgam concept, which was already taken up by Shulman (1987), must therefore possibly be seen in a larger framework and include aspects such as knowledge, skills, beliefs, etc., which have an effect on the learning performance of the students as a whole. As Santagata and Yeh (2016) have already shown, this also makes a “chain of effects” of individual variables difficult to differentiate.

A limitation of the paper is the somewhat small sample size in light of the extensive study design, but this is partly due to dropouts related to the COVID-19 pandemic. In addition, despite or perhaps because of the complexity of the video and text vignettes, the number of these items is relatively small, which may result in a loss of diagnostic accuracy, generalizability, and reliability (Blömeke et al., 2015b). In this regard, Brennan (2001, p. 295) refers to methodological challenges with the “reliability–validity paradox.” Thus, it can ultimately be assumed that the fundamental tension between validity and reliability cannot be eliminated in complex contextualized testing. The closer the survey is to the everyday situation with its indeterminacy and multidimensionality, the higher is the validity of the measurement on the one hand, but on the other hand the more losses have to be accepted with regard to its reliability. Furthermore, although the selected closed response formats with predefined options for action in the text and video vignettes have the advantage of economic data collection and analysis, in contrast to open response formats, no insights into ways of thinking and reasoning are provided. Also, no active production and construction of contexts of meaning is required. Adequate solutions therefore do not have to be produced, but merely assessed.

Finally, in the present study, various aspects of the cascade model, such as teaching actions, teaching quality, professional competencies in a broader sense, cognitive learning performance, etc., were not taken into account. In order to obtain a more comprehensive picture of possible interdependencies, corresponding aspects must be taken into account and integrated into future studies. The EPiC-PE project meets this demand and thus also opens the black box of teaching (see Blömeke et al., 2022). Corresponding studies and investigations on the interaction of teachers’ and students’ abilities (see Grönqvist & Vlachos, 2008) are currently in preparation.

## Supplementary Information

Sample Items CK-N, PCK-N, Basketball technique course

Sample Video Vignette

## Footnotes

1 Teachers’ CK predicts 10% of their students’ learning gains over a one-year period (Baumert et al., 2010).

2 PCK predicts nearly 40% of their students’ learning gains over a one-year period (Baumert et al., 2010).

99 * indicates significance with *p* ≤ 0.05; ** indicates significance with *p* ≤ 0.01; *** indicates significance with *p* ≤ 0.001.

## Declarations

All procedures performed in studies involving human participants or on human tissue were in accordance with the ethical standards of the institutional and/or national research committee and with the 1975 Helsinki declaration and its later amendments or comparable ethical standards. Informed consent was obtained from all individual participants included in the study.

## References

[CR2] Baumert, J., & Kunter, M. (2011). Das Kompetenzmodell von COACTIV. In M. Kunter, J. Baumert, W. Blum, U. Klusmann, S. Krauss & M. Neubrand (Eds.), *Professionelle Kompetenz von Lehrkräften. Ergebnisse des Forschungsprogramms COACTIV* (pp. 29–53). Waxmann.

[CR1] Baumert, J., Kunter, M., Blum, W., Brunner, M., Voss, T., Jordan, A., Klusman, U., Krauss, S., Neubrand, M., & Tsai, Y. M. (2010). Teachers’ mathematical knowledge, cognitive activation in the classroom, and student progress. *American Educational Research Journal*, *47*(1), 133–180. 10.3102/0002831209345157.

[CR3] Baumgartner, M. (2018). „… Kompetenz ohne Performanz ist leer! Performanz ohne Kompetenz blind …!” Zu einem integrativen Kompetenzstrukturmodell von Sportlehrkräften. *Zeitschrift für sportpädagogische Forschung*, *6*(1), 49–68. 10.18747/PHSG-coll3/id/514.

[CR4] Begle, E. G. (1972). *Teacher knowledge and student achievement in algebra*. School mathematics study group report, Vol. 9. Stanford University.

[CR5] Blömeke, S., Gustafsson, J.-E., & Shavelson, R. J. (2015a). Beyond dichotomies. Competence viewed as a continuum. *Zeitschrift für Psychologie*, *223*(1), 3–13. 10.1027/2151-2604/a000194.

[CR7] Blömeke, S., König, J., Suhl, U., Hoth, J., & Döhrmann, M. (2015b). Wie situationsbezogen ist die Kompetenz von Lehrkräften? Zur Generalisierbarkeit der Ergebnisse von videobasierten Performanztests. *Zeitschrift für Pädagogik*, *61*, 310–327. 10.25656/01:15350.

[CR6] Blömeke, S., Jentsch, A., Ross, N., Kaiser, G., & König, J. (2022). Opening up the black box: Teacher competence, instructional quality, and students’ learning progress. *Learning and Instruction*, *79*, 1–11. 10.1016/j.learninstruc.2022.101600.

[CR8] Bonß, W. (2001). Vom Theorie-Praxis-Problem zur Verwendungsforschung und wieder zurück. In T. Hug (Ed.), *Einführung in die Methodologie der Sozial- und Kulturwissenschaften* (pp. 91–102). Schneider.

[CR9] Brennan, R. L. (2001). *Generalizability theory*. Springer.

[CR10] Bromme, R. (1992). *Der Lehrer als Experte. Zur Psychologie des professionellen Wissens*. Huber.

[CR11] Bromme, R. (2008). Lehrerexpertise. In W. Schneider & M. Hasselhorn (Eds.), *Handbuch der Pädagogischen Psychologie* (pp. 159–167). Hogrefe.

[CR12] Büchel, S., Brühwiler, C., Egger, P., Hochweber, A. C., Kolovou, D., & Perret, J. (2022). Professionswissen von Sportlehrpersonen und Zusammenhänge mit motivationalen Orientierungen und Überzeugungen zum Lehren und Lernen im Sport. *German Journal of Exercise and Sport Research*, *52*, 558–569. 10.1007/s12662-022-00826-x.

[CR13] Cohen, J. (1992). Statistical power analysis. *Current Directions in Psychological Science*, *1*(3), 98–101. 10.1111/1467-8721.ep10768783.

[CR14] Depaepe, F., Verschaffel, L., & Kelchtermans, G. (2013). Pedagogical content knowledge. A systematic review of the way in which the concept has pervaded mathematics educational research. *Teaching and Teacher Education*, *34*, 12–25. 10.1016/j.tate.2013.03.001.

[CR15] Eisenberg, T. A. (1977). Begle revisited: teacher knowledge and student achievement in algebra. *Journal for Research in Mathematics Education*, *8*(3), 216–222. 10.2307/748523.

[CR16] Fishbein, M., Triandis, H. C., Kanfer, F. H., Becker, M. H., Middlestadt, S. E., & Eichler, A. (2001). Factors influencing behavior and behavior change. In A. Baum, T. R. Revenson & J. E. Singer (Eds.), *Handbook of health psychology* (pp. 3–17). Lawrence Erlbaum.

[CR17] Gogoll, A. (2020). Kompetenzorientierter Sportunterricht 2030 – Grundlagen für eine vernunftgetragene Selbstgestaltung des lebenslangen Sporttreibens. *Leipziger Sportwissenschaftliche Beiträge*, *61*(1), 51–67.

[CR18] Grönqvist, E., & Vlachos, J. (2008). *One size fits all? The effects of teacher cognitive and non-cognitive abilities on student achievement*. Institute for Labour Market Policy Evaluation.

[CR19] Hattie, J. (2009). *Visible learning: a synthesis of over 800 Meta-analyses relating to achievement*. Routledge. 10.4324/9780203887332.

[CR20] Hayes, A. F., & Krippendorff, K. (2007). Answering the call for a standard reliability measure for coding data. *Communication Methods and Measures*, *1*, 77–89. 10.1080/19312450709336664.

[CR21] Heemsoth, T., & Wibowo, J. (2020). Fachdidaktisches Wissen von angehenden Sportlehrkräften messen. *German Journal of Exercise and Sport Research*, *50*(2), 308–319. 10.1007/s12662-020-00643-0.

[CR22] Helmke, A. (2015). *Unterrichtsqualität und Lehrerprofessionalität: Diagnose Evaluation und Verbesserung des Unterrichts* (6th edn.). Klett.

[CR23] Herrmann, C., Seelig, H., Ferrari, I., & Kühnis, J. (2019). Basic motor competencies of preschoolers: Construct, assessment and determinants. *German Journal of Exercise and Sport Research*, *49*(2), 179–187. 10.1007/s12662-019-00566-5.

[CR24] Iserbyt, P., Ward, P., & Li, W. (2017). Effects of improved content knowledge on pedagogical content knowledge and student performance in physical education. *Physical Education and Sport Pedagogy*, *22*, 71–88. 10.1080/17408989.2015.1095868.

[CR25] Kahneman, D. (2011). *Thinking, Fast and Slow*. Penguin Books.

[CR27] Kaiser, G., Busse, A., Hoth, J., König, J., & Blömeke, S. (2015). About the complexities of video-based assessments: theoretical and methodological approaches to overcoming shortcomings of research on teachers’ competence. *International Journal of Science and Mathematics Education*, *13*, 369–378. 10.1007/s10763-015-9616-7.

[CR26] Kaiser, G., Blömeke, S., König, J., Busse, A., Döhrmann, M., & Hoth, J. (2017). Professional competencies of (prospective) mathematics teachers—cognitive versus situated approaches. *Educational Studies in Mathematics*, *94*(2), 161–182. 10.1007/s10649-016-9713-8.

[CR28] König, J. (2015). Kontextualisierte Erfassung von Lehrerkompetenzen. Einführung in den Thementeil. *Zeitschrift für Pädagogik*, *61*(3), 305–309.

[CR30] Krauss, S., Brunner, M., Kunter, M., Baumert, J., Blum, W., Neubrand, M., & Jordan, A. (2008). Pedagogical content knowledge and content knowledge of secondary mathematics teachers. *Journal of Educational Psychology*, *100*(3), 716–725. 10.1037/0022-0663.100.3.716.

[CR31] Krauss, S., Lindl, A., Schilcher, A., Fricke, M., Göhring, A., Hofmann, B., Kirchhoff, P., & Mulder, R. H. (Eds.). (2017). *FALKO: Fachspezifische Lehrerkompetenzen. Konzeption von Professionswissenstests in den Fächern Deutsch, Englisch, Latein, Physik, Musik, Evangelische Religion und Pädagogik*. Waxmann.

[CR29] Krauss, S., Bruckmaier, G., Lindl, A., Hilbert, S., Binder, K., Steib, N., & Blum, W. (2020). Competence as a continuum in the COACTIV study: the “cascade model”. *ZDM Mathematics Education*, *52*(2), 311–327. 10.1007/s11858-020-01151-z.

[CR32] Kunter, M., Klusmann, U., Baumert, J., Richter, D., Voss, T., & Hachfeld, A. (2013). Professional competence of teachers: effects on instructional quality and student development. *Journal of Educational Psychology*, *105*(3), 805–820. 10.1037/a0032583.

[CR99] Lüsebrink, I., Messmer, R., & Volkmann, V. (2014). Zur Bedeutung von Biografie, Erfahrung und Narration für die Fallarbeit in der Sportlehrer/innenausbildung. Zeitschrift für sportpädagogische Forschung, 2(1), 21-40. 10.5771/2196-5218-2014-1-21.

[CR33] Masters, G. (1982). A rasch model for partial credit scoring. *Psychometrika*, *47*(2), 149–174. 10.1007/BF02296272.

[CR34] Meschede, N., Fiebranz, A., Möller, K., & Steffensky, M. (2017). Teachers’ professional vision, pedagogical content knowledge and beliefs: on its relation and differences between pre-service and in-service teachers. *Teaching and Teacher Education*, *66*, 158–170. 10.1016/j.tate.2017.04.010.

[CR35] Messmer, R. (2018). What is the subject matter of physical education? *German Journal of Exercise and Sport Research*, *48*(4), 508–515. 10.1007/s12662-018-0531-2.

[CR36] Messmer, R., Brühwiler, C., Gogoll, A., Büchel, S., Vogler, J., Kruse, F., Wittwer, M., Steinberg, M., & Nadenbousch, A. (2022). Wissen und Können bei Lehrpersonen und Lernenden im Sportunterricht. Zum Design und zur Modellierung von Schüler*innen und Lehrer*innenkompetenzen. In R. Messmer & C. Krieger (Eds.), *Narrative zwischen Wissen und Können. Aktuelle Befunde aus Sportdidaktik und Sportpädagogik* (pp. 209–231). Academia. 10.5771/9783985720118-209.

[CR100] Miethling, W. D., & Giess-Stüber, P. (2007). Persönlichkeit, Kompetenzen und Professionelles Selbst des Sport- und Bewegungslehrers. In W. D. Miethling & P. Giess-Stüber (Eds.), *Beruf: Sportlehrer/in* (pp. 1-24). Schneider-Verl. Hohengehren.

[CR37] Muthén, L. K., & Muthén, B. O. (2017). *Mplus—Statistical analysis with latent variables—User’s guide* (8th edn.). Muthen & Muthen.

[CR38] Neuweg, G. H. (2014). Das Wissen der Wissensvermittler. Problemstellungen, Befunde und Perspektiven der Forschung zum Lehrerwissen. In E. Terhart, H. Bennewitz & M. Rothland (Eds.), *Handbuch der Forschung zum Lehrerberuf* (Vol. 2, pp. 583–614). Waxmann.

[CR39] Oslin, J. L., Mitchell, S. A., & Griffin, L. L. (1998). The game performance assessment instrument (GPAI): Development and preliminary validation. *Journal of Teaching in Physical Education*, *17*(2), 231–243. 10.1123/jtpe.17.2.231.

[CR40] Pill, S., Gambles, E.-A. F., & Griffin, L. L. (2023). *Teaching games and sport for understanding*. Routledge.

[CR41] Polanyi, M. (1966). *The tacit dimension*. University of Chicago Press.

[CR42] Putnam, R. T., & Borko, H. (2000). What do new views of knowledge and thinking have to say about research on teacher learning? *Educational Researcher*, *29*(1), 4–15. 10.3102/0013189X029001004.

[CR43] Ryle, G. (2009). *The concept of mind*. Routledge. 10.4324/9780203875858.

[CR44] Santagata, R., & Yeh, C. (2016). The Role of Perception, Interpretation, and Decision Making in the Development of Beginning Teachers’ Competence. *ZDM Mathematics Education*, *48*(1–2), 153–165. 10.1007/s11858-015-0737-9.

[CR45] Shulman, L. S. (1986). Those who understand: knowledge growth in teaching. *Educational Researcher*, *15*(2), 4–14. 10.3102/0013189X015002004.

[CR46] Shulman, L. S. (1987). Knowledge and teaching: Foundations of the new reform. *Harvard Educational Researcher*, *57*(1), 1–23. 10.17763/haer.57.1.j463w79r56455411.

[CR47] Tepner, O., & Dollny, S. (2014). Entwicklung eines Testverfahrens zur Analyse fachdidaktischen Wissens. In D. Krüger, I. Parchmann & H. Schecker (Eds.), *Methoden in der naturwissenschaftsdidaktischen Forschung* (pp. 311–323). Springer.

[CR48] Terhart, E. (2011). Lehrerberuf und Professionalität. Gewandeltes Begriffsverständnis – neue Herausforderungen. In W. Helsper & R. Tippelt (Eds.), *Pädagogische Professionalität* (pp. 202–224). Beltz.

[CR49] Vogler, J., Messmer, R., & Allemann, D. (2017). Das fachdidaktische Wissen und Können von Sportlehrpersonen (PCK-Sport). *German Journal of Exercise and Sport Research*, *47*(4), 335–347. 10.1007/s12662-017-0461-4.

[CR50] Vogler, J., Messmer, R., Wibowo, J., Heemsoth, T., & Meier, S. (2018). Drei Zugänge zur Modellierung fachdidaktischen Wissens von Sportlehrpersonen. In E. Balz & D. Kuhlmann (Eds.), *Sportwissenschaft in pädagogischem Interesse* (pp. 47–55). Czwalina.

[CR51] Ward, P. (2013). The role of content knowledge in conceptions of teaching effectiveness in physical education. *Research Quarterly for Exercise and Sport*, *84*, 431–440. 10.1080/02701367.2013.844045. 24592773 10.1080/02701367.2013.844045

[CR52] Wittwer, M. (2021). Fachwissen und Können von Sportlehrpersonen: Konstruktion eines Tests entlang des Kompetenzkontinuums. *Zeitschrift für sportpädagogische Forschung*, *9*(2), 60–77. 10.5771/2196-5218-2021-2-59.

[CR53] Wittwer, M., Messmer, R., & Büchel, S. (2023). Fachspezifisches professionelles Wissen und Können von Sportlehrpersonen. *Schweizerische Zeitschrift für Bildungsforschung*, *45*(2), 124–137. 10.24452/sjer.45.2.4.

[CR54] Yang, X., & Kaiser, G. (2022). The impact of mathematics teachers’ professional competence on instructional quality and students’ mathematics learning outcomes. *Current Opinion in Behavioral Sciences*. 10.1016/j.cobeha.2022.101225.

