# Supplementary material for: Effects of subject-specific professional knowledge and skills of physical education teachers on students’ learning progress
Source: Ger J Exerc Sport Res. 2024 Nov 8;55(4):659–71. doi: 10.1007/s12662-024-00992-0 (PMC12686084; doi:10.1007/s12662-024-00992-0)
Supplement: Supplementary file 1 — Sample Items CK-N, PCK-N, Basketball technique course [file 12662_2024_992_MOESM1_ESM.docx]

**Sample items CK**

**Table 1**

Sample items for assessing CK both as non-contextual knowledge with multiple-choice items and as contextual knowledge and skills with video vignettes in the area of TT

|  |  | **Paper-pencil method** | **Video vignettes** | |
| --- | --- | --- | --- | --- |
|  |  | **Non-contextual knowledge** | **Contextual knowledge and skills** | |
|  |  |  | **Movement analysis** | **Movement correction** |
| **CK** | **Knowledge of facts** | **Wie sieht ein technisch korrekter Korbleger im Basketball aus?**   1. Das letzte Dribbling erfolgt beim Aufsatz des Gegenfußes des Wurfarms. (*r*) 2. Nach der Ballaufnahme erfolgen noch drei Schritte bis zum Wurf. 3. Der Anlaufrhythmus lautet „jam-ta-tam“. (*r*) 4. Der Absprung erfolgt über das Bein, welches dem Wurfarm entspricht. 5. Der Ball sollte ohne Brettkontakt in den Korb fallen. | Sie sehen im Folgenden eine taktische Angriffsvariante im Handball. Es handelt sich dabei um ein Sperren-Lösen aus einer Körpertäuschung zur Wurfhandseite der ballführenden Spielerin. Schülerinnen einer 9. Klasse führen dieses Element im Rahmen einer 2:2-Situation aus. Die Ausführung der Schülerinnen ist zwar erfolgreich hat aber noch optimierungspotential.  **Welche verbesserungswürdigen technischen und taktischen Elemente erkennen Sie bei der ballführenden Angreiferin?**   1. Das Anprellen erfolgt nicht gerade aufs Tor, sondern zu fest nach außen (links). 2. Die Änderung der Laufrichtung (Täuschung) erfolgt mit zu großem Abstand zur Gegenspielerin. 3. Sie begeht Schrittfehler. 4. Die Änderung der Laufrichtung (Täuschung) sollte einbeinig erfolgen. 5. Die Seitwärtsbewegung vor dem Wurf erfolgt zu kurz. (*r*) 6. Sie fokussiert sich zu stark auf die Kreisläuferin und ist selber zu wenig torgefährlich. (*r*) | Welche Rückmeldungen wären Ihrer Meinung nach angebracht? Was wäre ein lernwirksames Feedback für die ballführende Spielerin?  **Schätzen sie die folgenden Rückmeldungen der Lehrperson zu den aufgeführten „Bewegungsmängeln" der Schülerin von 1 (gar nicht sinnvoll) bis 6 (sehr sinnvoll) ein!**  **Bewegungsmangel: Die Seitwärtsbewegung vor dem Wurf erfolgt zu kurz.**   1. Laufe nach dem Stoppschritt einen Schritt nach rechts und dann dynamisch mit dem linken Bein in Richtung Tor! (*6*) 2. Mache nach dem Stoppschritt zwei weitere Schritte in Richtung Tor! (*2*) 3. Mache den letzten Schritt in Richtung Tor mit dem linken Bein! (*5*) 4. Nutze den zur Verfügung stehenden Platz besser aus! (*4*) 5. Renne auf die Verteidigerin (Nr. 2) zu, damit diese aus dem Weg geht! (*1*) |
|  | **Reasoning** | **Weshalb sollten die Schüler im Basketball beim Korbleger von der linken Seite mit der linken Hand und beim Korbleger von der rechten Seite mit der rechten Hand werfen?**   1. Um den Ball vom Verteidiger abzuschirmen. (*r*) 2. Um den Ball aus einem besseren Winkel auf den Korb zu werfen. (*r*) 3. Um die Gefahr eines Schrittfehlers zu minimieren. 4. Um den Ball via Brett in den Korb werfen zu können. 5. Um die Gefahr eines Doppelfehlers zu minimieren. | **Weshalb sollte die ballführende Spielerin in der gezeigten Spielsituation optimalerweise einen zusätzlichen Schritt mit dem linken Bein Richtung Spielfeldmitte machen, bevor sie den Ball wirft bzw. ihrer Mitspielerin zupasst?**   1. Um eine größere Lücke zwischen den beiden Verteidigerinnen zu schaffen. (*r*) 2. Um die Torhüterin zu täuschen. 3. Um einen technisch korrekten (Sprung-)Wurf machen zu können und so mehr Torgefahr auszustrahlen. (*r*) 4. Um der Kreisläuferin mehr Zeit zu geben, um sich richtig zu platzieren. 5. Um ein Foul der Verteidigerin zu provozieren. | |

**Sample items PCK**

**Table 2**

Sample items for assessing PCK both as non-contextual knowledge with multiple-choice items and as contextual knowledge and skills with text vignettes in the area of TT

|  |  | **Paper-pencil method** | **Text vignettes** |
| --- | --- | --- | --- |
|  |  | **Non-contextual knowledge** | **Contextual knowledge and skills** |
| **PCK** | **Instructional strategies and representations** | **Sie beobachten beim Thema Handball, dass es vielen Schülern aus Ihrer 8. Klasse schwerfällt, mit dem Ball in Ziele zu treffen. Welche der folgenden Spielformen sind geeignet, um die Fertigkeit „ins Ziel treffen“ zu verbessern?**   1. Zwei Teams spielen gegeneinander. In der gegnerischen Endzone befinden sich Reifen. Punkte werden erzielt, wenn der Ball in einen Reifen in der gegnerischen Endzone niedergelegt werden kann, ohne dass ein Gegenspieler diesen durch hineinstehen blockiert. 2. Zwei Teams spielen gegeneinander. Jedes Team verteidigt einen Schwedenkasten. Punkte werden erzielt, wenn es gelingt den Ball mit einem Aufsetzer gegen eine der 4 Seiten des gegnerischen Kastens zu spielen. (*r*) 3. Zwei Teams spielen gegeneinander auf zwei Spielfeldhälften. Punkte werden erzielt, wenn ein Zuspiel ohne Gegnerberührung von einer Spielfeldhälfte in die anderen gelingt. 4. In einem Abstand von fünf Metern zur Wand werden zur Wand ausgerichtete Tore aufgestellt. Diese befinden sich in einem Torraum, der nicht betreten werden darf. Punkte werden erzielt, indem indirekt (über die Wand) Treffer erzielt werden. (*r*) 5. Zwei Teams spielen gegeneinander. Punkte werden erzielt, wenn ein ballführender Spieler einen Gegenspieler mit dem Ball berühren (abtupfen) kann. | **18 Schülerinnen, Thema: Basketball. Die Regeln sind den Schülerinnen bekannt.**  **Gegen Ende der Doppelstunde gibt es ein Basketballturnier. Die Lehrerin bildet vier ausgeglichene Gruppen. Auf zwei Spielfeldern können alle Teams gleichzeitig spielen. Als Schiedsrichterinnen stellen sich zwei Schülerinnen zur Verfügung. Schon bald stellt sich heraus, dass die Spielerinnen mit den Entscheidungen der Schiedsrichterinnen nicht immer einverstanden sind. Sie beschweren sich bei ihnen und wollen die Entscheidungen der Schiedsrichterinnen nicht akzeptieren. Die Schülerinnen protestieren bei jeder Gelegenheit. Die Lehrerin bricht das Spiel nach mehreren Protesten in beiden Spielfeldern ab.**  **Was würden Sie in der Rolle der Lehrerin jetzt sagen oder machen?**  **Wie schätzen Sie die folgenden Handlungsmöglichkeiten fachdidaktisch ein?**   1. Ich instruiere die Schülerinnen, dass immer diejenige Schülerin, welche einen Korb geworfen hat zum neuen Schiedsrichter wird. Diejenige Schülerin, die bis anhin Schiedsrichterin war, ersetzt deren Position. Somit übernimmt ständig eine andere Schülerin die Schiedsrichterrolle. (*5*) 2. Ich thematisiere mit der Klasse den Fairplay-Gedanken des Spielens. Anschließend lasse ich die Klasse ohne Schiedsrichterinnen spielen. Die SuS sollen sich so bei strittigen Situationen selbständig einigen. (*5*) 3. Ich wechsle die Schiedsrichterinnen aus und bestimme die beiden Spielerinnen als neue Schiedsrichter, die sich jeweils am stärksten beschwert haben. (*2*) 4. Ich erkläre den Schülerinnen, dass der Job der Schiedsrichterin nicht einfach ist und bitte sie um fairplay. (*4*) 5. Ich bespreche mit der Klasse nur zwei Regeln (Doppel- & Schrittfehler). Die Schiedsrichterinnen sollen sich im Folgenden nur auf diese beiden Regeln fokussieren. (*5*) |
|  | **Students (mis)conceptions & difficulties** | **In Ihrer 8. Klasse beobachten Sie beim Pick & Roll im Basketball, dass der gestellte Block vom Verteidiger einfach umgangen werden kann. Worauf könnte dieses Problem zurückgeführt werden?**   1. Zu schmaler Stand des Blockstellers. (*r*) 2. Zu langes zuwarten mit dem Ablaufen des ballführenden Angreifers nachdem der Block gesetzt wurde. (*r*) 3. Der ballführende Angreifer umgeht den Block mit zu großer Distanz. (*r*) 4. Fehlendes seitliches Verschieben des Blockstellers im Block, um den Verteidiger nicht passieren zu lassen. 5. Fehlendes aktives Wegdrücken des Verteidigers durch den Blocksteller mit den Armen. | **Thema Handball. Die Klasse besteht aus 12 Schülerinnen und 14 Schülern. Der Lehrer teilt die Klasse in 4 gemischte Teams. Aufgrund der Hallengröße können jeweils nur 2 Teams spielen, die anderen Schüler/innen sowie die Auswechselspieler/innen sitzen auf Bänken am Hallenrand.**  **Die Knaben spielen hauptsächlich untereinander, nur selten werden Mädchen angespielt. Diese sind nach einiger Zeit sauer und beschweren sich bei den Knaben. Daraufhin gehen einige Mädchen zum Lehrer und beschweren sich bei ihm über die Spielweise der Knaben.**  **Der Lehrer redet mit den Knaben: „Bezieht auch die Mädchen mit ins Spiel ein. Wir machen das jetzt so: Mädchen-Tore zählen doppelt.“ Die Knaben spielen jedoch nach wie vor nur untereinander. Mehr und mehr Mädchen lassen sich auswechseln oder verlassen einfach das Spielfeld und setzen sich auf die Bank.**  **Was würde Sie jetzt an Stelle des Lehrers unternehmen?**  **Wie schätzen Sie die folgenden Handlungsmöglichkeiten fachdidaktisch ein?**   1. Der große Teil der Klasse spielt wie gewünscht Handball. Deshalb lasse ich das Spiel wie bis anhin weiterlaufen. (*1*) 2. Die Knaben sollen sich während den nächsten fünf Minuten auf die Bank setzen und die Mädchen können unter sich spielen. Anschließend starten wir einen erneuten Versuch mit den gemischten Teams. (*1*) 3. Ich bilde zwei Teams mit je einem Mädchen- und einem Knabenblock. Somit spielen lediglich die Mädchen und die Knaben gegeneinander, werden aber von ihren Mitspielern des anderen Geschlechts unterstützt. (*6*) 4. Ich unterbreche das Spiel und thematisiere das Problem auf fachlicher Ebene. Diejenige Mannschaft, die alle Spieler ins Spiel mit einbezieht profitiert vermehrt von Überzahlsituationen. (*3*) |

**Items technique course basketball**

**Table 3**

Items for assessing sporting actions in order to measure student achievement.

| **Item** | **Description** | **Points** |
| --- | --- | --- |
| **Technik Korbleger rechts** | - Schrittabfolge und -rhythmus = letztes Prellen auf Gegengruß, 2 Schritte (rechts-links) *(zwingend!)* - Schwungbeineinsatz - Wurf einhändig via Brett mit der rechten Hand | - 0 = Technik falsch & kein Treffer, kein Korbleger (z.B. Standwurf) - 1 = Technik korrekt & kein Treffer oder Technik falsch & Treffer - 2 = Technik korrekt & Treffer |
| **Technik Korbleger links** | - Schrittabfolge und -rhythmus = letztes Prellen Gegengruß, 2 Schritte (links-rechts) *(zwingend!)* - Schwungbeineinsatz - Wurf einhändig via Brett mit der linken Hand | - 0 = Technik falsch & kein Treffer, kein Korbleger (z.B. Standwurf) - 1 = Technik korrekt & kein Treffer oder Technik falsch & Treffer - 2 = Technik korrekt & Treffer |
| **Technik Dribbling** | a) kontrolliertes & hüfthohes Prellen (max. 2 Dribblings über Höhe Bauch) –> Hand nicht höher als unterer Rippenbogen  b) Mit Außenhand prellen (Ball schützen) – Handwechsel im Slalom | - 0 = weder a) noch b) - 1 = a) oder b) sind erfüllt - 2 = a) und b) sind erfüllt |
| **Sprungstopp** | a) Pass (inkl. Stopp 🡪 paralleler Stand) aus dem Dribbling ohne Schrittfehler (≤ 2 Schritte)  b) Korrekter Sprungstopp (Ball in der Luft mit beiden Händen fangen, Füße berühren bei der Landung gleichzeitig den Boden) | - 0 = weder a) noch b) - 1 = a) ist erfüllt - 2 = b) ist erfüllt |
| **Druckpass** | a) Beidhändiger Druckpass auf Brusthöhe  b) gerade Flugbahn (max. 50cm über Kopfhöhe) | - 0 = weder a) noch b) - 1 = a) ist erfüllt - 2 = a) und b) sind erfüllt |
| **Ballannahme & Sprungstopp** | a) Ballannahme ohne Schrittfehler (≤ 2 Schritte)  b) Korrekter Sprungstopp: Ballannahme in der Luft, beidfüßig Landen, Orientierung zum Korb, anschließend keinen Schritt mehr;  Alternative: beidfüßige Landung vor der Ballannahme | - 0 = weder a) noch b) - 1 = a) ist erfüllt - 2 = b) ist erfüllt |
| **Stand- /Sprungwurf** | - Ball auf Wurfhand aufgelegt, Stützende Hand seitlich - Ellenbogen des Wurfarms zeigt zum Korb - Gleichzeitiges Strecken der Beine und des Wurfarms | - 0 = Technik falsch, kein Treffer - 1 = Technik korrekt, kein Treffer / Technik falsch, Treffer - 2 = Technik korrekt + Treffer |
